# Understanding Vietnam’s drug policy for amphetamine-type stimulants misuse

**DOI:** 10.1186/s12954-022-00621-9

**Published:** 2022-05-13

**Authors:** Mai Thi Ngoc Tran, Michael P. Dunne, Giang Minh Le, Hoe Dinh Han, Trang Thu Nguyen, Hai Thanh Luong, Quang Hung Luong, Ha Nguyen Pham, Philip Baker

**Affiliations:** 1 Faculty of Health, School of Public Health and Social Work, Queensland University of Technology, Brisbane, Australia; 2 Centre for Research and Training on Substance Abuse -HIV, Hanoi Medical University, Hanoi, Vietnam; 3 Nursing and Midwifery Faculty, Hanoi Medical University, Hanoi, Vietnam; 4 Australian Centre for Health Law Research, Queensland University of Technology, Brisbane, Australia; 5 Institute for Community Health Research, Hue University, Hue, Vietnam; 6 School of Social Science, The University of Queensland, Brisbane, QLD Australia; 7 School of Nursing, Midwifery and Social Work, The University of Queensland, Brisbane, QLD Australia; 8 Vietnam Union of Science and Technology Associations (VUSTA), Hanoi, Vietnam

**Keywords:** Drug policy, Amphetamine-type stimulants (ATSs), Law enforcement, Vietnam

## Abstract

**Introduction:**

The emergence of widespread amphetamine-type stimulants (ATSs) usage has created significant challenges for drug control and treatment policies in Southeast Asian countries. This study analyses the development of drug policies and examines current treatment program constraints in Vietnam to deal with ATS misuse. The aim was to gain insights that may be useful for national and international drug-related policy development and revision.

**Methods:**

A desk review of national policy documents and 22 in-depth key informant interviews were conducted from 2019 to 2021. Thematic content analysis was employed to identify key themes and their connections.

**Results:**

Analysis identified Vietnam’s 30-year history of developing policies and formulating strategies to reduce supply, demand, and harm from illicit drugs. With the increasing number of people who use ATS (PWUA), Vietnam has recently promoted harsh policy and law enforcement to deter drug use and supply. This policy trend prevails in many Asian countries. The three main constraints in dealing with ATS misuse emerged from punitive and restrictive drug policies. First, the general public believed that Centre-based compulsory treatment (CCT) is the only appropriate treatment for all types of illicit drug addiction despite its low-quality service provision. The rigid drug policy has led to social persuasion with impractical expectations for CCT effectiveness. Second, the emphasis on punishment and detention has hampered new drug treatment service development in Vietnam. CCT has become monopolistic in the context of impoverished services. Third, people who use drugs tend to hide their needs and avoid formal treatment and support services, resulting in declined social coherence.

**Conclusion:**

While new drugs are constantly evolving, the current law enforcement approach potentially constrains expertise to adopt effective treatment services. This study suggests that the top-down policing mechanism presently hinders the development of an appropriate intervention strategy for ATS misuse and diminishes social support to service providers.

## Introduction

Illicit drug usage patterns have changed significantly in recent years. The emergence of amphetamine-type stimulants (ATSs) in many countries is overtaking other drugs such as heroin, opium, and marijuana. According to the latest world drug report from the United Nations Office on Drugs and Crime [1, p. 79], there was an estimated 27 million people aged 15–64 using amphetamines. The rapid increase is often attributed to the low purchase cost. For example, in Southeast Asia, group usage (4 to 5 people) of methamphetamine costs collectively about $20–25 US [2].

Situated near the Golden Triangle of Laos, Myanmar, and Thailand, Vietnam has a long history of producing and utilising opium; however, ATS misuse is now widespread across Vietnam’s 63 provinces. According to a 2001 Government report, two-thirds of people who use drugs were dependent on heroin, 29.3% on opium, 1.5% used ATS, and 2.4% used other substances such as cannabis, cocaine, sedatives, and tranquillisers [3]. Since then, usage trends and patterns in Vietnam have changed from traditional to synthetic drugs [2]. At the end of 2020, an estimate of 190,000 people used ATS, which accounted for 80% of officially recorded illicit drug use, a sixfold increase since 2017 [4]. ATS is the preferred choice of new people who use drugs in large cities, with Methamphetamine (Vietnamese name: đá/Ice) becoming the most popular [4–6].

There are diverse political perspectives on illicit drug misuse prevention and treatment. Drug management strategies range from harsh penalties to reducing supply, incarceration of people who use drugs, compulsory treatment programs, to health promotion to increase community awareness about the harms of ATS to health and society [7]. Shi et al. [8] categorised four major drug control policies: complete prohibition, partial prohibition, depenalisation, and decriminalisation. Over the past two decades, as a “partial prohibition” country, Vietnam has supported advocacy to decriminalise personal drug use while also retaining harsh criminal punishment for trading drugs [9]. Earlier research had focused on analysing the transition of drug policy in Vietnam and identified the need to reduce sanctions [9–11]. However, most previous studies examined Vietnam’s drug law and policy development in general and have not investigated their effects on the development of effective treatment services. This study aimed to supplement existing research by investigating the transformations of drug policies in Vietnam and focused on existing treatment program constraints to examine the appropriateness of the laws and policies in the new context of ATS misuse.

This research study sought to explore:

1. How has drug policy evolved over recent decades in Vietnam?
2. How have the current drug laws and policies responded to people who use ATS?
3. What are the current treatment program constraints in dealing with ATS misuse in Vietnam?

## Methods

This study combines desk reviews and key informant in-depth interview analysis. We conducted desk reviews of relevant published drug policy manuscripts and legislative documents to understand the development of drug policies addressing illicit drug prevention and treatment in general and ATS specifically in Vietnam. We obtained those manuscripts by searching Medline (separately EBSCOhost and PubMed), Web of Science, and Google Scholar databases. Further, we retrieved the legal and regulatory documents from Vietnam’s legal databases (*thuvienphapluat.vn)* and the websites of Ministry of Health (MOH), Ministry of Labour, Invalids and Social Affairs (MOLISA), and Ministry of Public Security (MPS) in Vietnam. As a result, 25 relevant published manuscripts and 51 law and policy documents were included and analysed in full text in the Vietnamese language by two bilingual researchers.

Key informants (KIs) in-depth interviews were conducted in two rounds to improve understanding of drug policy implementation and reveal constraints regarding contemporary management of ATS misuse nationally. KIs were selected purposely based on the sample frame of key agencies suggested by the Addiction Treatment Network members in Vietnam. The sample inclusion criteria were: (1) participants were drug control policymakers or treatment specialists who had worked for at least five years within areas responsible for substance abuse law, policies, prevention programs, and treatment in Vietnam. Collectively, these participants were deemed to have broad knowledge about drug policies in Vietnam and recent experience working with people who use ATS; (2) participants held managerial roles in governmental or non-governmental organisations responsible for developing and implementing legal documents or providing services to people who use drugs in Vietnam. Twenty-three persons identified as key informants were invited by phone and email, of whom 22 agreed to participate in face-to-face interviews that were conducted from December 2020 to May 2021.

### Data analysis

Conventional thematic content analysis was employed to analyse the data following the approach of Razavi et al. [12]. Initial codes were developed based on the research questions and Walt and Gilson’s health policy analysis model, which included four elements: actors, process, content, and context. All documents and interview transcriptions were examined and then coded by the principal researcher with the support of NVIVO 12 software. To develop a systematic interpretation of the meaning of data, we cross-checked the legal documents, publications, and key informant interviews for consistency (data triangulation). Two members of the research team validated the analysis results.

We provided the KIs with the initial report in English and Vietnamese to gain feedback during the final report preparation. Critical feedback from the KIs on the draft report was discussed among the research team before inclusion in the report.

## Results

### The development of drug policy (1990–2021) and policy responses toward ATS misuse (Fig. 1)

Vietnam is a socialist country that emphasises the value of democracy and imbeds the rights of citizens in its constitution. The political foundation is one of unified democracy, reflected in the assertion of the leadership of the only ruling party, the Communist Party of Vietnam [13]. Many ministries are involved in drug prevention and treatment; however, primary responsibility belongs to three government ministries: Ministry of Health (MOH), Ministry of Labour, Invalids and Social Affairs (MOLISA), and the Ministry of Public Security (MPS). Much earlier, drug use and HIV was considered a dual epidemic in Vietnam as statistics showed that 60% of HIV-positive people who use drugs [14]. Drug usage was officially characterised as a social evil (Tệ nạn xã hội). This characterisation was embedded in the image of HIV disease, crime, and psychological and social derangement. PWUD is a socially undesirable group. Many were forced to engage in CCT treatment, even though most had no criminal conviction. Those addicted were labelled as “junkies” and “slaves of the drug” (thằng nghiện). Their images were portrayed in media with pictures of ghosts, skulls, cemeteries, and coffins [14].

The desk review revealed that drug policy was first seriously considered in the 1990s with the issuance of the Government’s Resolution 06/CP/1993 to prevent and fight against illicit drug use. It outlined the coordination of multi-sectoral agencies involved in drug use prevention. However, the main responsibility for drug treatment and social support for people who use drugs was assigned to MOLISA. People who use drugs admitted to compulsory treatment by the administrative and judicial systems had restricted freedom under this policy.

From 2000 to 2009, there were significant changes in drug policy due to increasing international funding for HIV/AIDS prevention and control in Vietnam [14, 15]. In 2008, methadone maintenance therapy was first trialled and then quickly scaled up nationally as one of Vietnam’s best evidence-based harm reduction approaches for people who use opioids [16, 17]. In 2009, Vietnam officially decriminalised drug use in criminal law, resulting in more harm reduction interventions via methadone clinics [18]. Consequently, there was a transition in drug prevention and control approach from “controlling social evils” to “increasing harm reduction treatment measures” [19]. In 2001, ATS was first listed as one of the “toxic narcotic substances” on Decree No. 67/2001/ND-CP. In 2004, UNODC implemented the first pilot project in Vietnam to improve the police’s interdiction and seizure of recognised ATS and their precursors [5]. However, no specific policies or Government programs for people who use ATS were established in this decade. People who were detected to use ATS were likely to be constrained in CCT centres [3].

The decade of 2010 to 2019 included several important movements regarding policies for people who use drugs, including those using ATS. With the support of international donors, Vietnam has avoided entanglement in the two-decade “war on drugs” in neighbouring Asian countries such as the Philippines, Thailand, and China [20, 21]. Methadone maintenance therapy was quickly scaled up nationally as the best evidence-based model for people who use opiates [16, 17]. In 2018, Vietnam had 55,000 people (about 24% of the estimated 226,000 total users) treated with methadone [22, 23]. This improvement was continued with another guiding document from MOH on ATS interventions by psychotherapies in 2019 (Decision No. 786/QD-BYT-2019). This was the first official Vietnamese policy document that provided an overall framework for ATS interventions, aligning with the WHO’s guidelines on screening drug misuse disorders and taking a harm reduction approach [24, 25]. However, this analysis revealed an absence of legal documents specifying State agencies’ responsibilities for implementing ATS interventions and treatment services. As a result, some harsh aspects of the management of drug dependence remained constant, and CCT centres and prisons continued the incarceration of many people who use ATS and other non-opioid drugs. In 2019, there were around 35,000 detainees in CCT centres (about 15% of the estimated 226,000 total users). Law enforcement and harm reduction approach continued to coexist in Vietnam [9].

Our legislation review identified that the Vietnam drug misuse prevention strategy involves controlling supply, demand reduction, and harm reduction targets. Although the process of reforming from a punitive approach to a harm reduction approach appears to have been effective, analysis shows this reform is recently becoming unfavourable to policymakers in the 2020s. The large quantity of seized drugs and the increasing number of people using ATS raised concerns about social issues with drugs. In August 2019, the Vietnamese Government shifted to strengthen policing and law enforcement to control drug misuse with the release of Directive 36 of the Central Committee of the Communist Party of Vietnam. There were many reasons for this policy reform. Firstly, as a prominent member of the Association of Southeast Asian Nations (ASEAN), Vietnam has taken seriously its responsibility to implement regional drug control policies. As such, it is now consistent with policies of most ASEAN countries to impose strict penalties on drug users who violate the law and thwart drug trafficking through the *Work Plans* to 2025, “Against illicit drugs in ASEAN” [10, 26]. There is a real concern that less strict laws may drive up ATS trafficking across borders [27] and national insecurity from ATS misuse [28]. Secondly, in the context of the withdrawal of international financial and professional advisory support, Vietnam must now draw on its resources to strengthen its policies toward PWUD. Policymakers appear to believe that effective addiction treatment does not always necessarily have to be voluntary. CCT system was established a long time ago and can accommodate a wide variety of addicts and social problems. Finally, like other Asian countries, the Vietnamese are affected by Confucianism, where collectivism and individual responsibilities to the group are highly valued. Drug use remains considered as a behaviour of irresponsible people [29, 30]. Decriminalisation could create uncertainty in law enforcement, and crime groups could capitalise on the ambiguity of legislative documents.

In early 2021, the National Assembly ratified a new drug prevention and control law with a high approval rate (95%), effective from 1 January 2022. Compared to the previous Laws,^1^ some harsh aspects of compulsory management of drug misuse remain constant in the 2021 drug law. For example, “*abstinence*” is still the sole legitimate therapeutic goal (Vietnam National Assembly, 2021). Furthermore, the new 2021 law supplements more regulations to increase drug use detection, including “*compulsory biological testing drug use*” and “*compulsory drug dependence screening*” for suspected people. Compulsory treatment eligibility has changed from 18 to just 12 years old. The 2021 law also classifies people who use drugs into two groups for management, dependent and non-dependent ones. However, provisions included in CCT centres are not because of identified drug dependence, and people who continue to use drugs and ignore alerts of the police and community authorities will be placed in the CCT centres. As a result, in 2020, the number of new detained people who use drugs in CCT centres surged to 55,480, with approximately 38,000 people detained for using ATS [31]. These numbers are nearly double that of 2018.

**Fig. 1:**
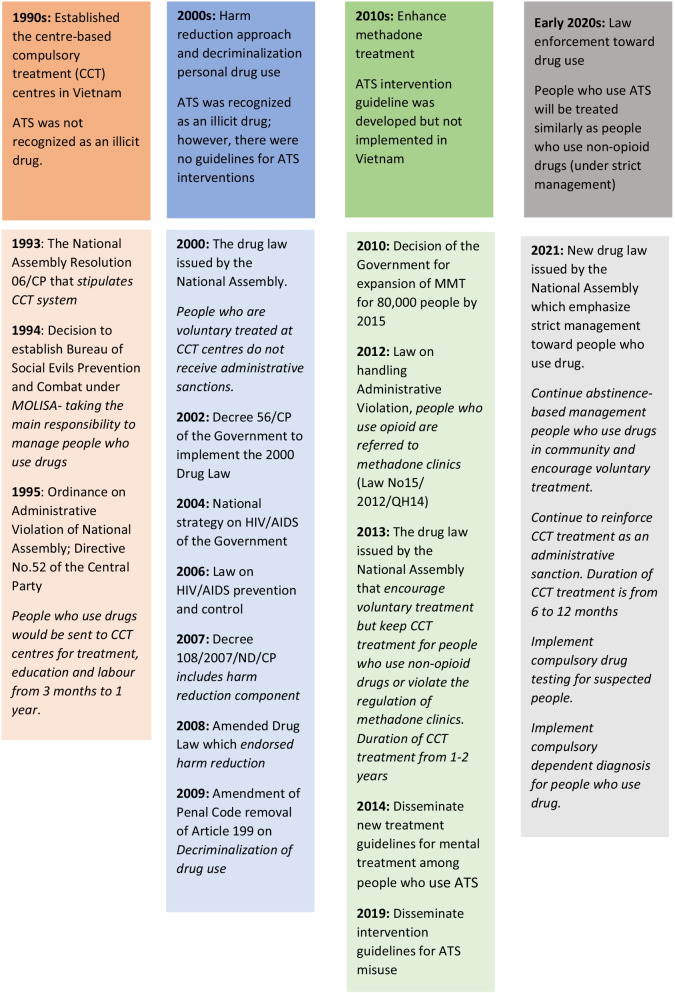
Milestones of drug policy development in Vietnam

### Policy implementation trends for the arrest, quarantine, and compulsory treatment of people who use ATS in Vietnam (Fig. 2)

The following analysis identified how Vietnam’s harsh laws are applied in detection, community management, and treatment of people who use ATS. This part aimed to investigate how policy responses to people who use ATS differ from those to heroin and opioids users.

**Fig. 2:**
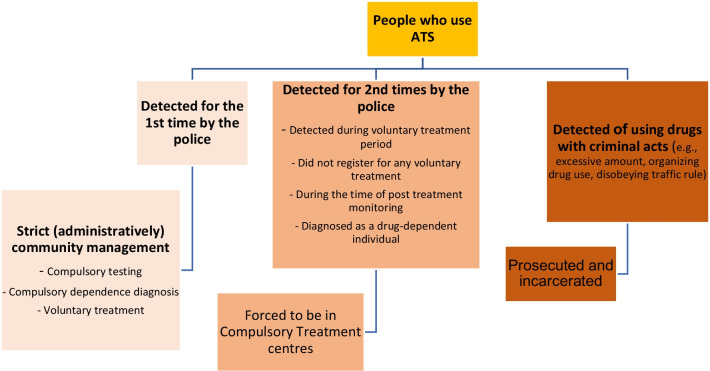
Managing people who use ATS following the Vietnam’s 2021 drug law

### Criminal punishment emphasis rather than administrative sanctions

The policy document review identified drug laws in Vietnam as a strategy of partial prohibition where drug trafficking and production are considered criminal violations but not for an individual’s drug use. People identified as people who use drugs through a positive test or caught during drug usage will be warned or fined between VND 500,000 (around $23 US) and 1,000,000 (around $46 US) for illegal narcotics, including ATS use.^2^ However, in practice, ATS usage is more easily detected by police than the usage of heroin or other opioids due to users’ observable behaviours, compulsory testing policy and methods of policing. These findings align with statements from an interviewee:

People who use ATS have a variety of recognised signs, and they can easily violate the criminal law because of ATS usage characteristics and the arresting circumstances. For example, such persons often gather to use drugs at motels and bars. So they are vulnerable to detection and violating the laws through organising illegal drug use or having a larger amount of drugs than the specified amount. (**KI from the MPS).**

KIs indicated that Vietnamese laws contain many provisions, which result in people who use ATS being susceptible to severe punishments rather than the more supportive provisions of administrative sanctions. Conviction and severity of sentencing appear to be influenced by the social setting of usage, which raises the risk that unrelated contextual factors can lead to imprisonment for use by individuals. The analysis reveals that there are risks for people who use ATS to passively violate the crime of organising, trading and storing illegal drug use^3^ or the transportation law as presented below by a KI:

Methamphetamine can remain in the body for up to 72 hours, so the user might still have a positive test three days after usage. If the person is included in a traffic accident, they are more vulnerable to a transportation law infringement and receive heavier penalties.^4^ Even the effect of the drug on mental health is not certified, they still have a potential of 3 to 10 years of imprisonment. (**KI from the MPS).**

### Community management policies for people who use ATS: limited early prevention and support, the dominance of control and quarantine

Although the laws have stipulated the responsibilities of each government ministry and social organisation in drug prevention, in practice, the responsibility for detecting and controlling people who use drugs and dealers mainly involves the police. The involvement and support for people who use drugs from the community organisations and local authorities vary in different localities. The legal documents also focus on the responsibility of departments in managing contact information and criminal records of people who use drugs, rather than looking for harm reduction’s intervention. When KIs were asked to discuss reasons for limited prevention and support to people who use ATS in residential areas, interviewees explained as below:

At the community level, there is no officer with full-time responsibility for illicit drug prevention. People who use ATS are managed at the community level by local police officers. The local authority manages people who use ATS and those who use other drugs based on the information of the police. There is limited support from police for social programs that aim to reduce drug use at the community level. If People who use drugs cannot stop using drugs themselves, they will be sent to compulsory centres. **(KI from the Centre for Disease Control (CDC) at the provincial level**).

In Vietnam, the prevention of ATS misuse was often understood as early detection of people who use ATS and the enforcement of immediate abstinence. Quarantine was considered by many KIs to be a suitable prevention solution at the community level. However, this contradicts the 2019 guidelines on ATS interventions which call for service responses to support people who use ATS who need psychological treatment and social care. Paradoxically, when informants described Vietnamese drug prevention policy development, they mainly emphasised the restriction and control over people who use ATS rather than psychological and social interventions:

Policymakers, as well as the general population, believe that quarantine is a good drug prevention strategy. By early detection, quarantining people who use ATS in the community, compulsory centres or jails can save the lives of many other people in our society. (**KI from National Committee on HIV/AIDS, Drugs and prostitution prevention and control -NCADP).**

To summarise, arrest and punitive control strategies are predominant both in policies and law enforcement in Vietnam. There is still inadequate prioritisation of effective prevention and support activities.

### ATS misuse treatment policy: limited voluntary services, a compulsory tendency

The document analysis identified the presence of two treatment streams for people who use drugs in Vietnam, including ATS: voluntary and compulsory stream. The laws and policies mention both treatment streams, with a preference towards enhancing voluntary treatment and reducing compulsory treatment.^5^ However, there was little evidence in documents or interviews that voluntary treatment stream is actually favoured by regulators over compulsory drug treatment. Instead, the primary purpose of encouraging voluntary treatment appears to emphasise people who use drugs’ responsibility to pay part of their treatment costs.

If detainees (people who use drugs in the centres) come here as being compulsory by police’s referral, the State will cover 100% of the cost, and if they are voluntary (family or community member- referred), they will pay 30% and the State will support 70%. (**KI from CCT Centre**).

The concept of “*voluntary treatment*” in legal documents is understood as self-compliance with the state regulations. From the interviewees’ perspectives, it appears that drug use behaviours are considered to be primarily the *“fault of the failure of individuals”.* Social determinants, including networks around people who use drugs, are not regarded as significant influences, and therefore, treatment programs tend not to focus on improving social support. An individualistic attitude prevails; it is expected that people who use drugs should try to stop using drugs by themselves before being coerced, as explained by this informant:

People who use illicit drugs are not the victims. They are the ones who take the initiative of using drugs. This idea is also the general perception of lawmakers in Vietnam, which strongly influences the regulation content and treatment availability. The responsibility of drug addiction treatment first belongs to PWUD and their families. This perception is accepted by most people in our society (**KI of National Assembly**).

This analysis revealed a shortage of voluntary treatment services for people who use ATS. With Government subsidies, methadone clinics appear the sole system that provides voluntary treatment, but they currently only accept people who use opioids, not those who use ATS. At the same time, though CCT centres can offer voluntary treatment to people who use ATS with Government funding, few centres have attracted voluntary people who use ATS in practice. As mentioned above, the existing national guideline for ATS treatment only stipulate general directions without specific implementation mechanisms or propose practical models for people who use ATS. As a result, people in Vietnam who use ATS are in reality, most often forced to be in compulsory treatment.

### Constraints of prevention and treatment for ATS misuse in Vietnam (Fig. 3)

The following three main themes emerged during our data triangulation from key informant interviews and policy documents analysis.

**Fig. 3:**
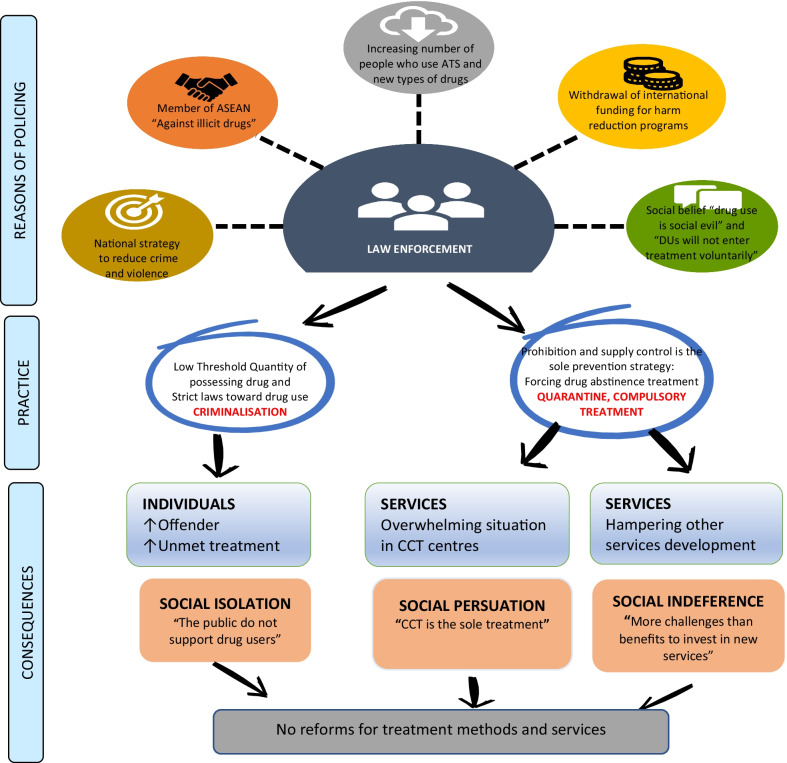
Law enforcement in Vietnam

### An overwhelming situation in compulsory centres: high cost, low effectiveness

The increasing detection and arrest of ATS misuse have led to a rapid increase in the number of people in CCT. This escalation has resulted in a heavy, unsustainable workload for CCT staff, leading to poor quality services for detainees. A key informant who had more than 20 years of management experience in CCT centres described the current overwhelming situation:

The number of people who use ATS has increased rapidly, accounting for over 70% of the centres’ detainees. This centre can only accommodate up to 300 detainees, but we always have above that number, sometimes up to 650 people. The accommodation rooms are insufficient, and the dining room is not enough to meet their basic needs. Counselling and other treatment rooms are also limited. (**KI from CCT centre**)

Substance use treatment has been assigned to MOLISA, while the capacity to supply services at CCT and the community level remains limited. For example, with only 120 centres nationally in a population of over 96 million people, most local communities have no access to services for ATS misuse. However, according to the new 2021 drug law, people should be forced into CCT centres if they continue using drugs, though many may not be dependent users. Key informants interviewed in this study stated that compulsory treatment is necessary, but it should only be prioritised for a small number of violent and dangerous people who use drugs. KIs were concerned about the high cost of compulsory treatment:

The state budget, which is allocated to directed detainees, is also quite large. However, investment in running the facilities is much bigger. Hanoi has 7 CCT centres, and the State spends about 200 billion VND (around USD 8.8 million) a year on this system to serve about 3,000 detainees. (**KI from CCT centre**)

In the legal documents of Vietnam, the two concepts “people who use drugs” (nguoi su dung ma tuy) and “people who are dependent on drugs” (nguoi nghien ma tuy) has just been clarified in the new drug law 2021. However, it remains the condition of forcing CCT treatment for people who continue to use illegal drugs and do not adhere to the abstinence rule. In Vietnam, the rate of people who use ATS at a high risk of dependence is under 10%. But because of various circumstances, we are putting non-dependent users into CCT centres and building a cumbersome program. We do not have enough money to “punish” so many people, including new users who violate the regulation of voluntary treatment (**KI from UN Agency**).

Thus, the system-wide responses to the ATS epidemic should be more sophisticated. At present, people who use ATS have not been categorised in the legal system by levels of dependence or harm they pose to the community. They tend to be lumped together and receive the same therapies and length of stay at CCT centres, which is believed to create more harm to them.

The consequences of detention and enforced treatment are not only the cost lost of the State but also the impact on the desperate life of people after they leave CCT centres. Many arrested people because of ATS misuse had to go to compulsory centres while they were employed. After being released from CCT, they re-enter society with guilt and discrimination, and it is easy for them to relapse (**KI from UN Agency**).

The effectiveness of the CCT system was criticised by many interviewees, and it is worth noting that the shortcomings were mainly blamed on the inadequate scale of the system. The key informants from CCT centres did not accept the low quality of service as a major problem. For example, when asked about the high risk of relapse after ATS misuse treatment from CCT centres, he argued that:

The relapse rate in Vietnam is not different from other countries in South East Asia, around 60%. When the detainees are released, they are followed up by phone calls once a month in the next six months. Every month, the staff here make a phone call to ask about their situation. However, the rate of response is meagre; they do not listen or answer very quickly. In here, we did not only provide treatment. We have to manage and control people who use drugs to stay here, not go out of the centre (**KI from CCT centre)**

The expressed attitude does not necessarily reflect a lack of compassion but rather the social beliefs many service providers have held for a long time. These conversations show that coercion is a barrier to developing a therapeutic relationship and voluntary treatment. The CCT system has existed in Vietnam for many years, reflecting the State’s strict control over drug use, including ATS use, though it has not stimulated treatment quality improvement.

### Underdeveloped voluntary treatment services: the shortage of services and low readiness of service providers for ATS misuse treatment

As mentioned above, the number of people who use ATS in need of treatment is high, and public services such as CCTs are overcrowded. This situation is exacerbated by the limited availability of private health and social services for people who use ATS in Vietnam. One KI succinctly summarised the contemporary situation of private addiction treatment:

Currently, there are only 16 private addiction treatment centres in the whole country that have been provided with licenses for their operation. But by 30 May 2020, only 1,103 people who use drugs (0.004%) had been treated in private centres since they opened. Each year, the number of people who use ATS being treated in these facilities is only a few hundred. (**KI from MPS**)

The interviews proved several obstacles to the development of new addiction treatment services. For example, working with people who use ATS is challenging as staff are often concerned about being victims of crime. In addition, there are complicated legal requirements for establishing private treatment services as there is a high risk of financial loss. Moreover, the number of people who use ATS in Vietnam has increased rapidly across diverse socio-demographic groups. Similar to other countries, we found that many users in Vietnam are from affluent families. Yet, there appeared to be a strong opinion amongst policymakers that users usually have low socio-economic status, so they cannot afford treatment without government support. One KI affirmed as below:

For other health services in Vietnam, if the Government could not meet the needs, the private sector will jump in immediately for benefits. However, services for people who use drugs could not develop without the Government’s subsidy. Generally, people do not want to work with people who use drugs, but that is our social responsibility. The risks to opening a private addiction treatment centre in Vietnam are much bigger than the benefits. In 2019 and 2020, there were no new registered services for drug addiction treatment (**KI from the MoH**).

Additionally, several pilot interventions showed the effectiveness of services that integrate ATS misuse treatment in methadone clinics in Vietnam. Unfortunately, this approach has not been scaled up and new treatment methods for people who use ATS are not promoted in MMT system in Vietnam.

At methadone clinics, there was no legal mechanism for us to treat people who use ATS. Our responsibility is to treat people who use opioid drugs in the daytime only. People who use drugs have too many social problems, and we cannot manage them. The arresting of a patient outside our clinic due to ATS use resulted in treatment interruption. But we cannot expand new services even though having the Ministry of Health’s technical guidelines for ATS treatment in 2019 because of Article 10, Decree 90^6^**(KI from MMT clinic).**

Among the various challenges of establishing new services for people who use ATS, KIs indicated that criminal punishment of people who use ATS is the most significant barrier for service development. The analysis identified that punishment and arrest are inhibitors for service development and also disrupt or hinder treatment.

### People who use ATS hide their needs and avoid formal treatment and support services

Given the large increased number of people who use ATS in the past decade, it should be predicted that many more people who use ATS would attend services, at least for legal, counselling and health care support. Unfortunately, this did not occur. With an estimate of 190,000 people who use ATS in 2020, the recorded number of people who use ATS presenting at formal support services remained very low:

People who use ATS are exposed to many risks, and they need both health and legal advice. But in Vietnam, they would not go to a specialist to be advised because they are scared. Following the report of MOLISA in 2020, the number seeking help due to Amphetamine misuse in 2020 is just 976 people at all 16 private services in Vietnam (**KI from the MPS).**

Low service use may be a direct consequence of harsh criminalised policies. Many policymakers expect that harsh regulations can prevent people from using drugs and reduce drug-related crime. However, the harsh regulations seem to drive drug use further underground. People who use ATS tend to hide their needs for support services and treatment programs because they are afraid of being detected by authorities and forced to compulsory treatment. In many cases, they may eventually end up in detention. Also, there is suspicion of treatment effectiveness in CCT centres among many people who use ATS. Many fear official recognition of their problems because of the stigma and shame felt by families, employers, and social networks. Together, these factors prevent users from seeking help. These concerns are illustrated by comments from an interviewee:

Every day, when examining patients, we know a lot of them have used methamphetamine. However, they refuse to discuss the effects of methamphetamine on their physical and mental health. That is because they were afraid of being sent to the CCT centre and not receiving further treatment at the MMT facility and afraid of the stigma of family members and people surrounding them **(KI from the MMT clinic).**

People who solely depend on opioids can be referred to methadone clinics; however, people who use ATS or other non-opioid drugs could be treated strictly in CCT centres or are placed under restrictive community management. Therefore, they often hide and do not seek help.

There are escalating concerns that people who use ATS are involved in the drug trade and therefore implicated in serious crimes. The borderline between a non-trading user of ATS, who should not be arrested and detained, and a drug dealer, who will be arrested, is often unclear to police and others in the judicial system. Thus, for many people who use ATS, it seems safer to withdraw themselves from being recognised by society:

Under Vietnamese law, drug trafficking can lead to heavy penalties from imprisonment to execution. However, high profitability attracts many people who use drugs to be involved in trafficking. As a result, drug dealers are extremely desperate and get prepared to fight to protect their lives. Gradually, they split apart and form their own worlds, resulting in reduced social cohesion. (**KI from the MPS**).

## Discussion

Despite policies that favour decriminalisation of personal drug use in many parts of the world, in Vietnam and some other Asian countries, there remains strong opposition to liberal, harm reduction laws and regulations. The number of people in Vietnam who were arrested or forced into CCT centres because of illicit drugs has increased sharply in the past year (in 2019: 38,000 new cases in CCT, 22,400 arrested cases; in 2020: 55,480 new cases in CCT, 25,582 arrested cases) [32, 33]. This study shows that poor service supporting people who use ATS combined with more rigorous law enforcement has restricted access to suitable treatment for people who use ATS and forced many users into detentions. ATS misuse treatment services have been hampered because of many factors, including service providers’ fear of breaking the law by providing support to people who use ATS. In the context of the fast-growing number of people with ATS misuse in a new and challenging health and social situation, drug prevention and treatment reforms have mostly not changed in a positive direction. Indeed, the harm reduction initiatives in the previous two decades in Vietnam have faltered, with limited access to inclusive, non-judgemental services. Many people who use ATS fear being arrested and incarcerated. Evidence from our interviews and documents analysis suggests that many policymakers and service providers believe that this shift to a harsher policy regime is a challenge for service providers to deal with ATS misuse.

In Vietnam, the harsh regulations of law enforcement toward people who use ATS include a low threshold quantity for possession of illegal drugs (under 0.1-g heroin or methamphetamine), abstinence-based, compulsory treatment, and more recently compulsory testing and screening for drug dependence. Following prior research into the disadvantages of punishment and compulsory treatment [16, 34, 35], we now discuss the current treatment program constraints in dealing with ATS misuse in Vietnam, as well as in other countries with similar sociopolitical contexts.

### Emphasising abstinence-based compulsory treatment

Although CCT centres have many shortcomings [36], they continue to be used for publishment and control of people who use ATS. The preference for abstinence-based treatment has solid political and regulatory support. Under Vietnam's current legal system, abstinence-based treatment is still considered the utmost goal of drug misuse treatment.

The singular preference for state-based, compulsory treatment has created the social belief that these centres are valuable places for all types of drug misuse, with no exception for the complex and diverse needs of people who misuse ATS. Like Cambodia, people who use drugs often receive a referral through several pathways, such as police and family members, to join the CCTs for treatment [37]. Although the limits of CCT are acknowledged by some policymakers, there appears to be little effort to address the shortage of high-quality ATS treatment services. People who use ATS continue to be sent to CCT with impractical expectations of recovery.

Abstinence-based and compulsory treatments are no longer effective methods for stemming the flow of illegal synthetic drugs or reducing the number of people who use drugs, so drug control initiatives by attracting users are essential. Firstly, evidence shows that the drug market is dynamic and difficult to distinguish between illegal and legal drugs. A wide range of synthetic drugs is available today that is not included in the UNODC’s list of prohibited substances. UNODC publishes hundreds of substances and precursors each year but has been unable to identify any specific category of these substances [38]. So practical solution is to encourage users to register for services that will allow them to voluntarily report and be actively involved in controlling drugs and stimulants available on the market. Secondly, no pharmacological or psychosocial treatment can help people who use ATS quit using them entirely until now [39]. Thus, the relaxation of regulations on treatment management, such as abstinence-based treatment, is necessary. Aside from making the management of people who use drugs easier, this also allows the service providers to coordinate their responsibilities concerning new illicit substances that hit the market.

### Lack of private and voluntary services and social indifference toward new service development

Despite the heavy strain on current services caused by the influx of people who use ATS, there are few new options for treatment and social care. This problem is not specific to Vietnam, as other countries with punitive drug policies also have undeveloped private and voluntary treatment services. There were only 16 private drug misuse treatment centres in Vietnam by 2019, which accommodated just 0.004% of recorded people who use drugs (about 1000 among 260,000 recorded people who use drugs), even though the Government officially supported the provision of more private and voluntary services. Similarly, China had only 66 voluntary detoxification institutions nationwide, providing services for just 3030 drug users among 717,000 recorded drug users [40]. Clearly, there is not enough attention to more voluntary treatment services to meet the needs of such a vast number of people who use ATS.

King et al. [41] reported that around 10% of patients in their study in Vietnam had interrupted treatment because of incarceration and compulsory treatment.

The four pillars of drug prevention and control have been identified as law enforcement, prevention, treatment, and harm reduction [42]. However, our findings in Vietnam suggest that focusing too much on law enforcement might suppress the development of the remaining three pillars. Therefore, the rapid development of care and treatment services should be a top priority in rebalancing these pillars and managing users in the long run. In the current context of new types of drugs rapidly developing, with more than 80% of people who use ATS are not dependent [31], Vietnam has not had a clear policy to promote the development of drug misuse treatment services. Policymakers are affected by the provisions of law and the social views on ATS misuse treatment, and they hesitate when making decisions for more investment in ATS misuse treatment in Vietnam. Retention of a client in treatment depends on both their eligibility for legal treatment and the acceptance of their continued use of illicit drugs while in treatment.

## Conclusion

The increasing number of people who use ATS is alarming throughout the country. Arguably, implementing strict, zero-tolerance policies that emphasise harsh punishment is a significant barrier in the development of quality services for ATS misuse. While CCT centres are criticised for their low quality and overcrowding, private facilities and voluntary treatment services remain underdeveloped. It is critical that the Government pays more consideration to ATS prevention and treatment, both in legislative documents and practical working mechanisms, to establish more service providers and facilitate better coordination among relating agencies. In addition, there should be specific provisions for ATS prevention and treatments in the new drug law and its guiding decrees.

## Limitations

There are some limitations in this study that should be addressed in future research. First, the 22 experts participants in interviews were senior managers and experts who generally operate at provincial or national levels. Therefore, their reflections may not fully capture recently community-based issues regarding ATS prevention and control. Second, to explore the challenges faced by stakeholders in Vietnam in drug prevention and treatment, we may have overlooked some of the less apparent benefits of policy implementation.

## Footnotes

1 The Law on Prevention and Control of Narcotic Substances 2000, revised in 2008 and The Law on Prevention and Control of Narcotic Substances in 2013.

2 The Decree No.167/2013/ND-CP (Clause 1, Article 21) dated November 12, 2013, indicates that people who use drugs as well as people who use ATS should be sanctioned with administrative measures as the first priority.

3 Article 255 of the Penal Code 2015, amended in 2017, stipulates the crime of organising illegal drug use. If there are more than two people using drugs together, the organiser can be sentenced from 2 to 7 years in prison. Further, offenders can be imprisoned up to 15 years if participants include children from 13 to 18 years old, pregnant women, or people who are using detoxification methods. In cases where drug use badly affects the health and life of those who use together, the organisers could face life imprisonment. Article 250 of the Penal Code defines the crime of trading and storing illegal drugs. For example, persons who transport heroin, cocaine, amphetamine, methamphetamine, MDMA, or XLS-11 weighing from 0.1 to 5 g are to be sentenced from 2 to 7 years of imprisonment.

4 Clause 11, Article 5, Decree 46/2016/ND-CP, drivers with a positive test of an illegal substance will be deprived of the right to use their driving license from 22 to 24 months (in the case of having a driving license) or receive a fine of between VND 16 million and VND 18 million (in the case of not having a driving license or having one but being deprived of the right to use the driving license). Particularly, Clause 1 and Clause 2 of Article 260 of the Penal Code 2015 (and amended by Clause 72, Article 1 of the Penal Code Amendment in 2017) stipulates that people who have a positive drug test and cause a traffic accident which affects the health or life of others could be punished from 3 to 10 years of imprisonment.

5 The directive No. 25/2017/CT-TTg by the Vietnamese Prime Minister emphasised the application of administrative sanctions for persons committing acts of illegal use of narcotics.

6 Article 10, Decree 90/2016/ND-CP: People who were detected to have positive result test with other drugs different to opioids, will be suspended from treatment at MMT clinic.
